# The mitochondrial genome of the firefly, *Pyrocoelia amplissima* (Olivier, 1886) (Coleoptera: Lampyridae) and its phylogenetic analysis

**DOI:** 10.1080/23802359.2025.2468753

**Published:** 2025-07-09

**Authors:** Mao Wang, Lei Wang, Chunyan Yi, Jian Tang, Qingdong Chen, Zhenzhen Wei, Jingwei Guo, Yang Yang, Song Chen

**Affiliations:** Institute of Plant Protection, Sichuan Academy of Agricultural Sciences, MOA Key Laboratory of Integrated Management of Pests on Crops in Southwest China, Chengdu, China

**Keywords:** Lampyridae, *Pyrocoelia amplissima*, mitochondrial genome, phylogeny

## Abstract

We presents the first complete mitochondrial genome of *Pyrocoelia amplissima* (Olivier, 1886) (Coleoptera: Lampyridae: Pyrocoelia), a Chinese endemic firefly. The 17,426 bp circular genome contains 13 PCGs, 23 tRNAs, 2 rRNAs, and a control region with a notable A + T bias (76.91%). Phylogenetic analysis of 12 firefly species using 13 PCGs places *P. amplissima* basally within its genus. This complete mitochondrial genome provides valuable genetic information for further research on Lampyridae.

## 1. Introduction

**Figure 1.**
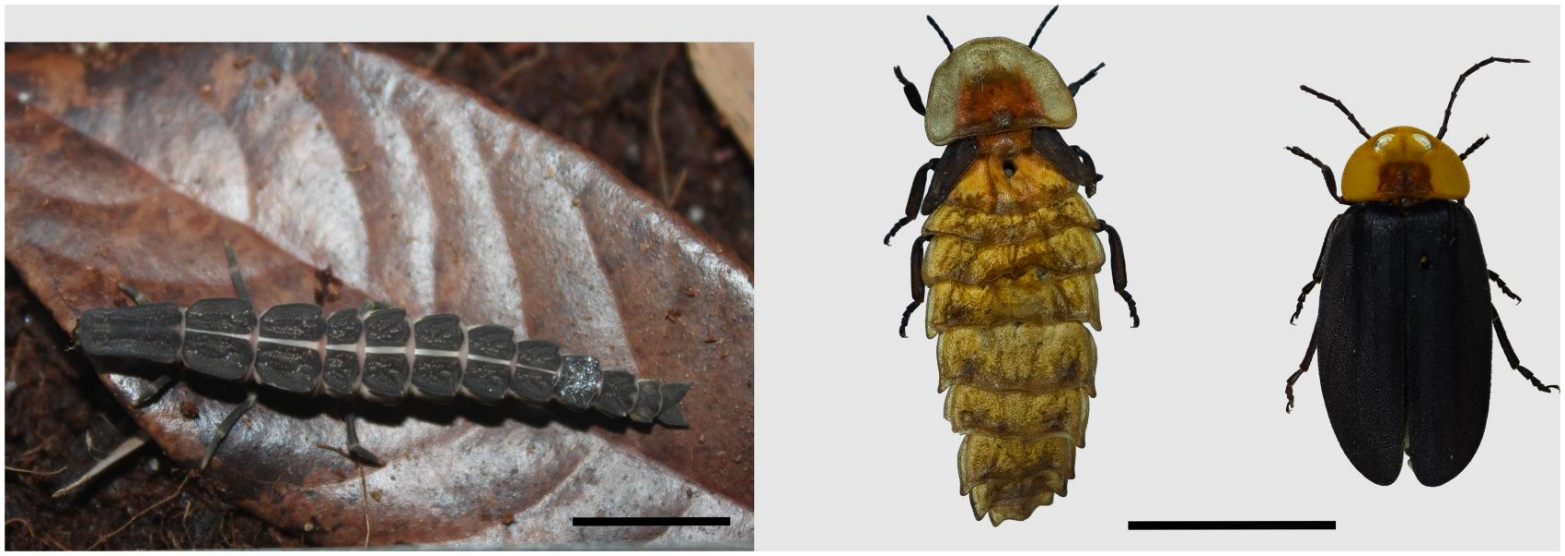
The specimen of *Pyrocoelia amplissima* (Olivier, 1886), (a) larval, scale bar = 10 mm. (b) adult, female on the left, male on the right, scale bar = 10 mm. Photographed and processed by Mao Wang.

Fireflies (Coleoptera: Lampyridae) rank among the most charismatic beetles, with distinctive bioluminescent courtship displays that make them a potential flagship group for insect conservation. With more than 2000 species worldwide, firefly beetles exhibit surprisingly diverse life history traits (Lloyd and Gentry 2009; Lewis 2016). *Pyrocoelia* Gorham, 1880 is a genus of terrestrial fireflies that thrives in humid forest environments. The larvae of this genus are notable for their predatory behavior, specifically targeting land snails (Osozawa et al. 2015). Species in this genus exhibit sexual dimorphism, with males being winged, while females possess vestigial elytra and lack hind wings (Jeng et al. 2011; Zhu et al. 2022) (Figure 1). *Pyrocoelia amplissima* (Olivier, 1886) (Coleoptera, Lampyridae, Pyrocoelia) is a beautiful ornamental insect endemic to China (including in Sichuan, Hubei, Yunnan, Chongqing, Guangxi, Fujian) (Fu 2014; Zhu et al. 2022). Typically, during the larval stage, *P. amplissima* is challenging to distinguish from other fireflies in the genus *Pyrocoelia*, which comprises over 60 known species (McDermott 1966; Jeng et al. 1999; Zhu et al. 2022). In this context, mitochondrial genome sequences are crucial for accurately distinguishing and classifying these larval stages (Lee et al. 2003; Wang et al. 2007). Currently, the complete mitochondrial genomes of four species in the genus *Pyrocoelia* are available on NCBI: *Procoelia rufa* AF452048; (Bae et al. 2004),; *Pyrocoelia analis* OK323960 (Ji and Xu 2022), *Pyrocoelia praetexta* NC_044790; (Chen et al. 2019), and *Pyrocoelia thibetana* NC_044792; (Chen et al. 2019). Previous studies on this species are very limited, and no genetic research has been conducted on *P. amplissima*. In this study, we sequenced the complete mitochondrial genome of *P. amplissima* to provide essential baseline data for a deeper understanding of phylogenetic relationships and to support future research within the Lampyridae family.

## 2. Materials and methods

### 2.1. Sample collection and preservation

The insect specimens were collected from Qianwei County, Leshan City, Sichuan province, China, on July 21, 2023 (altitude: 465 m, 29°23′6.40″N, 103°56′21.65″E). The submitted sample was a field-collected male adult that had undergone starvation treatment (Figure 1). The 24-hour starvation treatment was applied to reduce sample contamination for mitochondrial genome sequencing. During this period, the larvae were maintained under controlled conditions: temperature 20–25 °C, relative humidity 60–80%, and a 12-hour light/dark cycle. In terms of morphological characteristics, *P. amplissima* has a body length of 18–25 mm, which is larger than ordinary fireflies. The orange-red abdomen, orange-yellow pronotum, and orange coxa and femur, contrasted with the blackish remainder of the body, are key features for distinguishing it from other species within the same genus. (Zhu et al. 2022). The specimens were stored in alcohol at the institute of Plant Protection, Sichuan Academy of Agricultural Sciences, Chengdu, Sichuan, China (specimen numbers: No. FF2024041201; contact person: Mao Wang, 501812830@qq.com).

### 2.2. DNA extraction sequencing and genomic assembling

**Figure 2.**
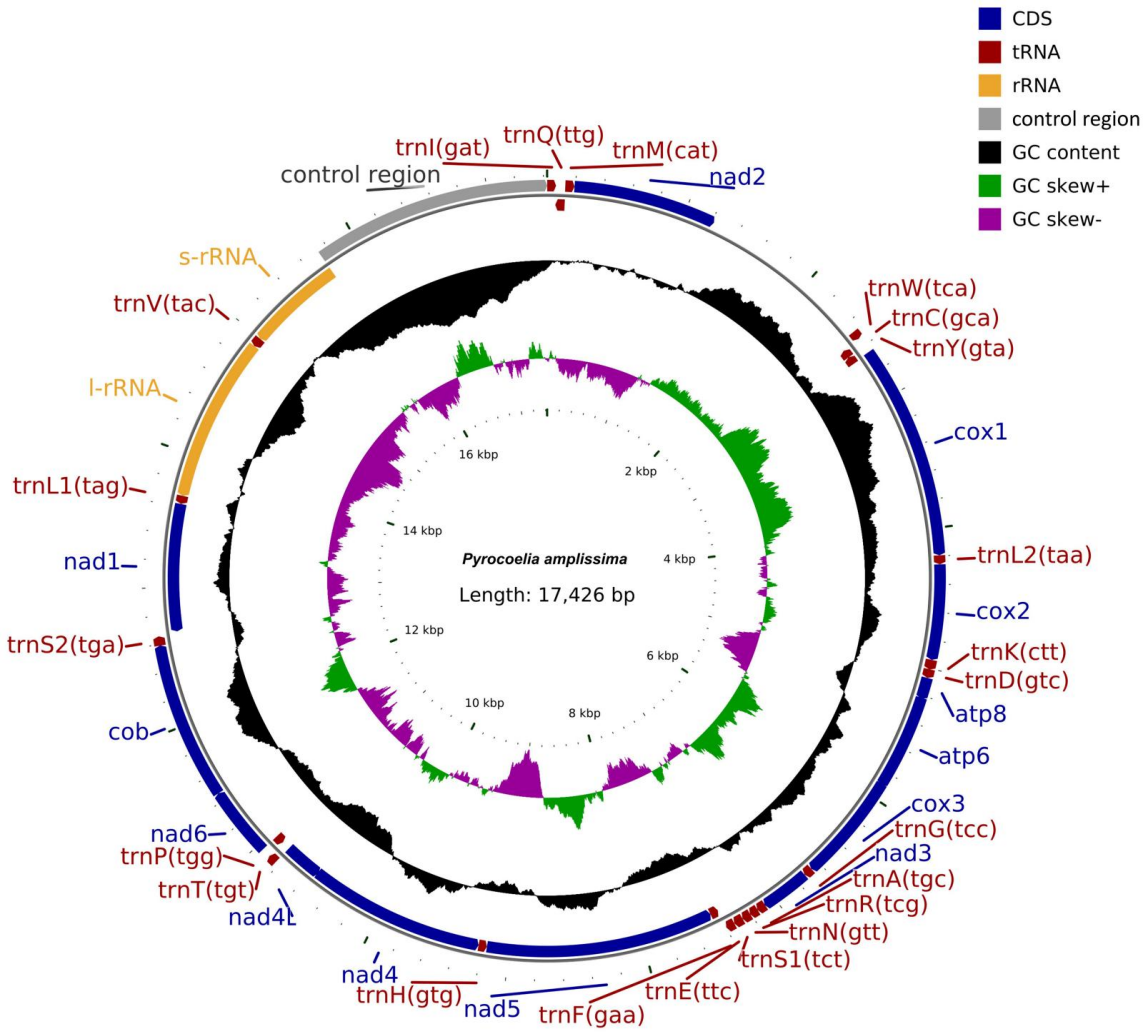
The circular-mapping mitochondrial genome of *Pyrocoelia amplissima*: the total length was 17,426 bp, which was divided into 37 genes, including 13 PCGs, 22 tRNAs, two rRNAs.

Genomic DNA was extracted from a male adult using the modified cetyltrimethylammonium bromide (CTAB) method (Doyle 1987). Sequencing of the complete mitogenome of *P. amplissima* was performed by Novaseq xplus in Shanghai Personalbio Technology Co., Ltd. (Shanghai, China), with a total data of 5 G (150 bp reads). Raw data quality control was conducted using Fastp v0.23.1 (Chen et al. 2018). The coverage-depth map is provided in Supplementary Figure S1. High-quality second-generation sequencing data were initially compared to the NCBI nt database using blastn (BLAST v2.2.31+) to identify potential mitochondrial sequence. Mitochondrial circular sequences were then assembled using GetOrgannelle v1.7.7.0 (https://github.com/Kinggerm/GetOrganelle). The final complete mitochondrial genome was refined using Pilon v1.18 (Walker et al. 2014), and subsequently annotated using the MITOS web server (http://mitos.bioinf.uni-leipzig.de/) (Bernt et al. 2013). In addition, the complete mitochondrial genome map was drawn using CGView (Stothard and Wishart 2005) (Figure 2).

### 2.3. Phylogenetic analysis

The phylogenetic tree was constructed using PhyloSuite 1.2.3 (Zhang et al. 2020) based on concatenated nucleotide sequences of 13 PCGs of *P. amplissima.* Twelve mitochondrial reference genomes representing two subfamilies and five genera of Lampyridae were downloaded from NCBI. Additionally, one species from the Rhagophthalmidae family was selected as the outgroup. Each coding gene was aligned individually using MAFFT v.7.313 (Katoh and Standley 2013), with codon alignment mode applied. Ambiguously aligned regions were removed using Gblocks v.0.91 (Castresana 2000) with default settings. Maximum likelihood (ML) phylogenies were inferred using IQ-TREE v2.2.0 (Nguyen et al. 2015), employing the optimal model identified by ModelFinder (Kalyaanamoorthy et al. 2017). Phylogenetic support was assessed with 1,000 ultrafast bootstrap replicates (Minh et al. 2013).

## 3. Results

### 3.1. Characteristics of P. amplissima genome

The complete mitochondrial genome sequence of *P. amplissima* (GenBank PP935118) has 17, 426 bp long and has a base composition of A (43.02%), C (13.89%), T (33.89%) and G (9.21%) (Figure 2 and Table S1). The A + T content of the whole *P. amplissima* mitogenome is 76.91% indicating a strong AT bias (Eyre-Walker 1997). The AT-skew and GC-skew of the mitogenome are 0.119 and −0.202, respectively (Table S1). In addition, the sequence includes 13 protein-coding genes, 22 transfer RNA genes, 2 ribosomal RNA genes, and a control region measuring 1,695 bp, characteristic of a typical insect mitochondrial genome (Wolstenholme 1992) (Figure S2). All 13 PCGs initiated with ATN (ATG, ATT, ATC, ATA and TTG) codon. Among those genes, five PCGs start with ATG (*apt6*, *cox3*, *nd4*, *nd4L*, *cob*), three PCGs initiate from ATT (*cox1*, *atp8*, *nd5*), two PCGs initiate from ATC (*nd2, nd6*), two PCGs start with ATA (*cox2*, *nd3*), and one PCGs start with TTG (*nd1*). In addition, the stop codons of the PCGs include five TAA (*nd2*, *atp8*, *atp6*, *nd4L*, *nd6*), and one TAG (*nd1*), whereas an incomplete terminal codon namely single T was found in seven PCGs (*cox1*, *cox2*, *cox3*, *nd3*, *nd5*, *nd4*, *cob*) (Table S2).

### 3.2. Phylogenetic position

**Figure 3.**
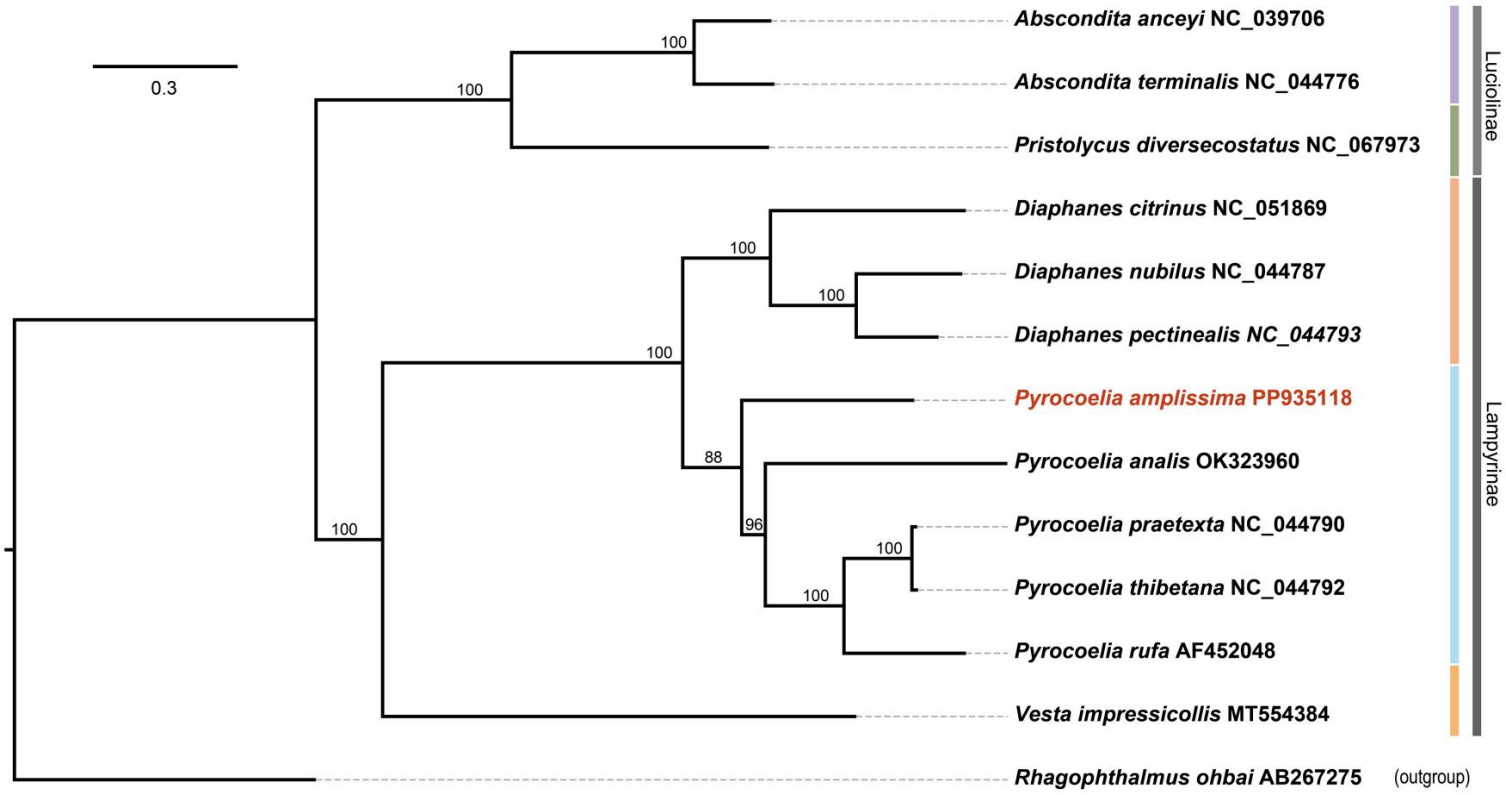
The maximum likelihood (ML) tree of *P. amplissima* and twelve other firefly species based on the concatenated 13 PCGs. The NCBI accession numbers of the mitochondrial genomes used for tree construction are listed as follows: *Diaphanes citrinus* NC_051869 (Yang and Fu 2019)), *Diaphanes nubilus* NC_044787 (Chen et al. 2019), *Diaphanes pectinealis* NC_044793 (Chen et al. 2019), *Pyrocoelia rufa* AF452048 (Bae et al. 2004), *Pyrocoelia analis* OK323960 (Ji and Xu 2022), *Pyrocoelia praetexta* NC_044790 (Chen et al. 2019), *Pyrocoelia thibetana* NC_044792 (Chen et al. 2019), *Vesta impressicollis* MT554384, *Abscondita terminalis* NC_044776 (Chen et al. 2019), *Abscondita anceyi* NC_039706 (Hu and Fu 2018), *Pristolycus diversecostatus* NC_067973, *Rhagophthalmus ohbai* AB267275 (Li et al. 2007).

To assess the phylogenetic relationships of *P. amplissima*, we downloaded the complete mitochondrial DNA sequences of eight Lampyridae species and three Luciolinae species, with *Rhagophthalmus ohbai* designated as the outgroup. The results indicated that *P. amplissima* is positioned basally within the genus *Pyrocoelia* and the *Pyrocoelia* and *Diaphanes* being more closely related to each other than to Vesta. Additionally, Lampyrinae is a sister group to Luciolinae. Within Lampyrinae, the species of *Pyrocoelia* and *Diaphanes* are grouped together within their respective clades, forming a sister group with *Vesta*, which is consistent with previous phylogenetic studies (Ji and Xu 2022) (Figure 3).

## 4. Conclusions and discussion

In this study, we report for the first time the complete mitochondrial genome of *P. amplissima* and present a detailed annotation (Figure 1). The genera *Pyrocoelia* and *Diaphanes* share similar morphological, ecological, and ethological characteristics, making them difficult to distinguish (Chen et al. 2019; Zhu et al. 2022). Our mtDNA phylogeny tree clearly separates them into two distinct clades (Figure 3), consistent with previous COX1-based studies (Zhu et al. 2022), but not with 16S and the concatenated genes (Li et al. 2006; Chen et al. 2019). This could be due to the inclusion of more morphologically ambiguous species in the latter analysis. Within the *Pyrocoelia* genus, *P. amplissima* has a broader distribution than most other species, which are primarily localized endemics (Zhu et al. 2022). Its basal position in the phylogenetic tree, combined with the hypothesis that the Oriental region is the origin of the genus (Li et al. 2006), suggests that *P. amplissima* plays a crucial role in shaping the evolutionary trajectory of *Pyrocoelia*. Therefore, much more phylogenetic studies incorporating morphological, molecular, and ethological data are essential. Overall, this study provides useful genetic information to accurately classify species, and offering a further understanding of the evolutionary relationship of *Pyrocoelia*.

## Supplementary Material

TableS1.xls

FigureS2.pdf

Figure_S1.pdf

TableS2.xls

## Data Availability

The genome sequence data that support the findings of this study are openly available in GenBank of NCBI at (https://www.ncbi.nlm.nih.gov/nuccore/PP935118.1) under the accession no. PP935118.1. The associated BioProject, SRA, and Bio-Sample numbers are PRJNA1153363, SRR30429269, and SAMN43381919 respectively.
