# Supplementary figures and images for: The mitochondrial genome of the firefly, Pyrocoelia amplissima (Olivier, 1886) (Coleoptera: Lampyridae) and its phylogenetic analysis
Source: Mitochondrial DNA B Resour. 2025 Jul 9;10(8):678–82. doi: 10.1080/23802359.2025.2468753 (PMC12243008; doi:10.1080/23802359.2025.2468753)

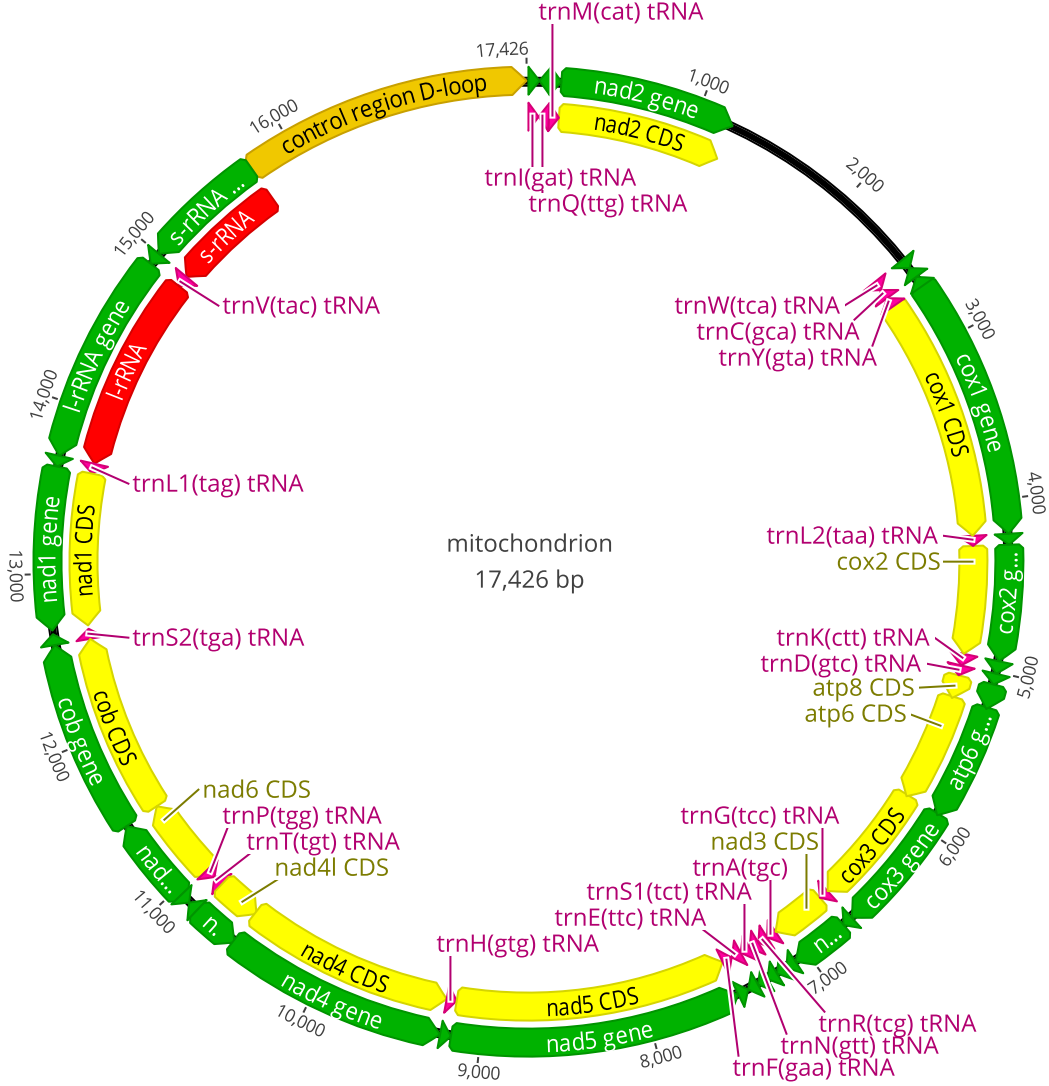

Supplement: FigureS2.pdf [file TMDN_A_2468753_SM6492.pdf]

*Pyrocoelia amplissima*

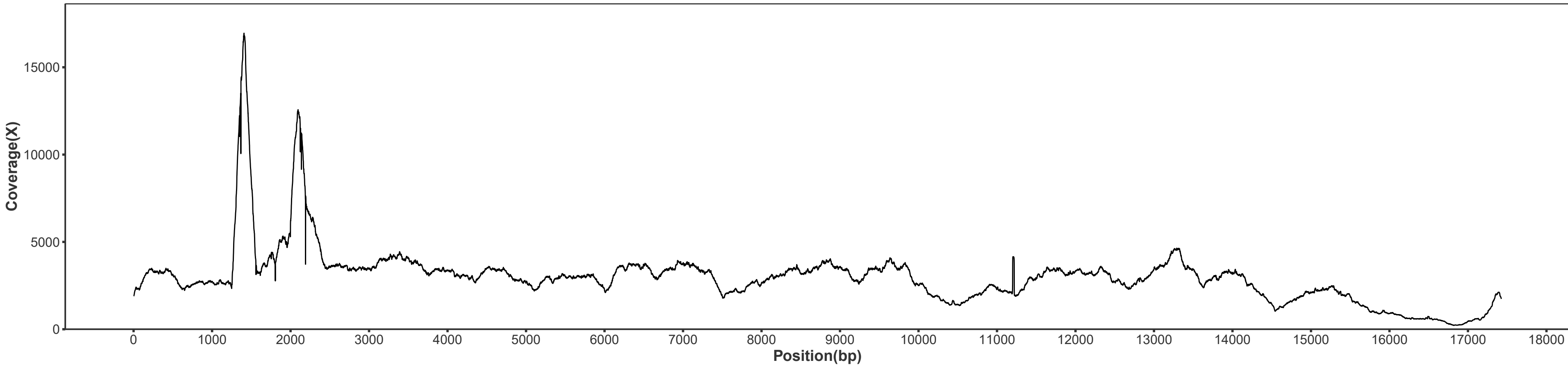

Supplement: Figure_S1.pdf [file TMDN_A_2468753_SM6491.pdf]
